# Nd-Fe-B Magnets: The Gradient Change of Microstructures and the Diffusion Principle after Grain Boundary Diffusion Process

**DOI:** 10.3390/ma12233881

**Published:** 2019-11-24

**Authors:** Yaojun Lu, Shuwei Zhong, Munan Yang, Chunming Wang, Liuyimei Yang, Longgui Li, Bin Yang

**Affiliations:** 1 Faculty of Materials Metallurgy and Chemistry, Jiangxi University of Science and Technology, Ganzhou 341000, Chinacmwang87@jxust.edu.cn (C.W.); 2 ARC Research Hub for Computational Particle Technology, Department of Chemical Engineering, Monash University, Clayton, Victoria 3800, Australia; liuyimei.yang@monash.edu; 3 DMEGC Magnetics Co., Ltd., Ganzhou 341000, China; lilongguiht@163.com

**Keywords:** Nd-Fe-B magnets, grain boundary diffusion, diffusion path, coercivity mechanism, corrosion resistance

## Abstract

The diffusion of Tb in sintered Nd-Fe-B magnets by the grain boundary diffusion process can significantly enhance coercivity. However, due to the influence of microstructures at different depths, the coercivity increment and temperature stability gradually decreases with the increase of diffusion depth, and exhibit good corrosion resistance at a sub-surface layer (300–1000 μm). According to the Electron Probe Micro-analyzer (EPMA) test results and the diffusion mechanism, the grain boundary and intragranular diffusion behavior under different Tb concentration gradients were analyzed, and the diffusion was divided into three stages. The first stage is located on the surface of the magnet, which formed a thick core-shell structure and a large number of RE-rich phases. The second stage is located in the sub-surface layer, forming a uniform and continuous RE-rich phase and thin core-shell structure. The third stage is located deeper in the magnet, and the Tb enrichment only existed at the triangular grain boundary.

## 1. Introduction

Sintered Nd-Fe-B magnets with an excellent coercivity have been widely used in the field of rail transportation, new high-energy automobiles, wind power generation, and other high-tech fields [1,2]. With the development of the downstream application field, higher requirements are put forward for the magnetic properties and service performance of sintered Nd-Fe-B magnets [3,4]. Heavy rare earth (HRE), such as Dy and Tb, are important elements to improve coercivity and service stability. In particular, the addition of Dy and Tb by the grain boundary diffusion process (GBDP) can not only effectively reduce the consumption of elements, but also avoid the reduction of remanence [5,6].

GBDP is an important method to repair microstructures and improve coercivity by using HRE on the surface of the magnet to diffuse along the grain boundaries [7,8]. Marko et al. [9] diffused TbF_3_ powders through the grain boundaries, forming a uniform core-shell structure and increasing the coercivity by 50%. Chen et al. [10] showed that the diffusion of the low-melting alloy, DyF_3_, along the parallel c-axis was better than that in the vertical direction, which was conducive to improving magnetic properties and reducing HRE consumption. Sueptitz et al. [11] found that the diffusion of DyF_3_ was conducive to the passivation of magnets under acidic conditions, showing better corrosion resistance. Komuro et al. [12] obtained an improvement in coercivity and lowered the loss of remanence through the Dy-F diffusion coating. However, the magnetic properties and microstructure of GBDP magnets at various positions are different due to the diffusion process of HRE elements. Zhou et al. [13] found that the magnetic properties and microstructures of the Dy diffusion magnet showed a gradient trend with the change of diffusion depth. Understanding the diffusion process of HRE and improving the diffusion depth and uniformity of elements are key points to solve the above problems and promote the development of GBDP to a larger scale.

This paper studies the effect of the Tb_2_O_3_ grain boundary diffusion on the magnetic properties and service characteristics of each part of the magnet. Based on the microstructure of the magnet, the main phase structure, and elements distribution, the diffusion process of Tb elements was studied, and the coercivity strengthening mechanism was revealed.

## 2. Materials and Methods 

Commercial sintered Nd-Fe-B magnets (N-48) were used with a size of 30 mm × 30 mm × 7 mm. The surface of the magnet was polished with 2000 grit SiC paper and then cleaned with alcohol in an ultrasonic cleaner. The Tb_2_O_3_ powder was mixed together with a reducing agent and alcohol, and then coated on the surface of the magnet perpendicular to the c-axis. The coating weight of the mixture accounted for 4% of the total weight of the magnet. Then, the diffusion heat treatment of the magnet was conducted at 950 °C for 10 h and 500 °C for 2 h, respectively.

**Figure 1:**
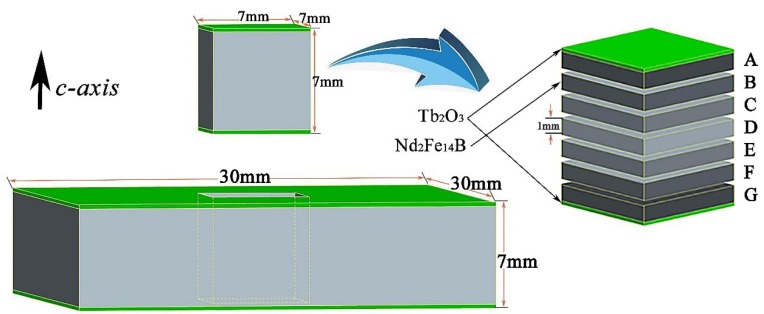
7 small magnetic blocks cut along diffusion depth.

Small blocks of 7 mm × 7 mm × 7 mm from the center of the magnet were selected and cut into 7 pieces along the diffusion direction, and were named from A to G successively, as shown in Figure 1. The magnetic properties of magnetic blocks were measured by a high-temperature permanent magnet measuring instrument (NIM-500C, National Institute of Metrology, Beijing, China). The magnetic properties of magnets with various diffusion depths at different temperatures were measured by the comprehensive physical property measurement system (PPMS-DynaCOOL1-9, Quantum Design, San Diego, CA, USA). The Tafel curves at different diffusion depths of the magnets in 3.5 wt % NaCl solution were determined by the standard three-electrode electrochemical workstation (CHI760e, CH Instruments, Austin, TX, USA). The phase structure of the magnets at various diffusion depths were analyzed by the X-ray Diffractometer (XRD-Empyrean, PANalytical B.V., Holland, The Netherlands). The microstructure of each diffusion layer was determined using the Scanning Electron Microscope (SEM-MLA650F, FLIR Systems, Inc., Wilsonville, OR, USA), and the distribution of elements at different microscopic regions was characterized by the Electron Probe Micro-analyzer (EPMA-JXA-8230, Japan Electronics Co., Ltd., Tokyo, Japan). In order to obtain these results at different depths of magnets, several sets of Tb_2_O_3_ diffusion magnets with the size of 7 mm × 7 mm × 7 mm were prepared and their surfaces were ground down to a certain thickness with emery papers in the experiment. 

## 3. Results and Discussion

The changes of magnetic properties at 300 K after Tb_2_O_3_ diffusion are shown in Table 1. The coercivity (*H*_cj_) of the magnet increased from 1180 kA·m^−1^ to 1685 kA·m^−1^ after diffusion treatment. The remanence (*B*_r_) and maximum magnetic energy product ((*BH*)_max_) of the Tb_2_O_3_ diffused magnet were 1.395 T and 347.98 kJ·m^−3^, respectively. There was no significant decrease compared with that of the original magnet (1.407 T and 351.48 kJ·m^−3^). It is worth mentioning that different materials and preparation methods could lead to different results. In previous studies, *H*_cj_ also realized better results with the electrophoretic deposition of submicron TbF_3_ [5,14]. These results also indicate that Tb_2_O_3_ coating on the magnets and heat treatment of the magnets at high temperatures could not only effectively improve *H*_cj_, but also avoid the adverse effects of Tb addition on *B*_r_ and (*BH*)_max_.

**Figure 2:**
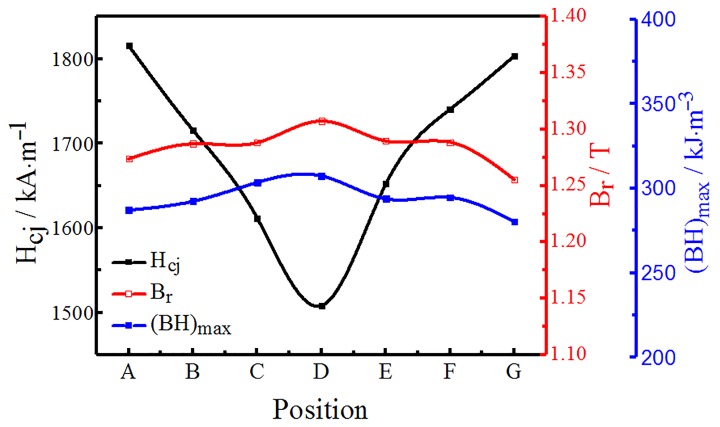
The magnetic properties of GBDP magnets at various diffusion depths.

At 300 K, the magnetic properties of the GBDP magnets at various diffusion depths were tested, as shown in Figure 2. With the change of diffusion depth, the *H*_cj_ of the magnet changed significantly; the highest *H*_cj_ was obtained on the surface (locations A and G), while the lowest *H*_cj_ was in the middle (location D), which shows a symmetric distribution. The *H*_cj_ of A and G were 1816 kA·m^−1^ and 1804 kA·m^−1^ respectively, while the *H*_cj_ in the middle position D was only 1509 kA·m^−1^. The difference of *H*_cj_ between the surface and the center of the magnet was as high as 307 kA·m^−1^. The *H*_cj_ of D was also higher than original magnet, which proves that Tb has diffused into the center and existed more in the grain boundary phase (Nd-rich) than the original magnet. Diffusion also caused symmetrical changes in *B*_r_ and (*BH*)_max_; the Tb diffusion magnets had a higher concentration at the surface area, which enhanced the higher anisotropy field phase (*H*a) and also led to the deterioration of *B*r and (*BH*)_max_. The *B*r and (*BH*)_max_ increased with the increase of the diffusion depth, but their changes were very slight. The above results prove that the element, Tb, on the surface of the magnet mainly diffused through the grain boundary, existed in the main phase and the grain boundary phase, and reduced the *B*_r_ and (*BH*)_max_ [2,15]. As the diffusion depth increased, the Tb became rarer in the main phase.

**Figure 3:**
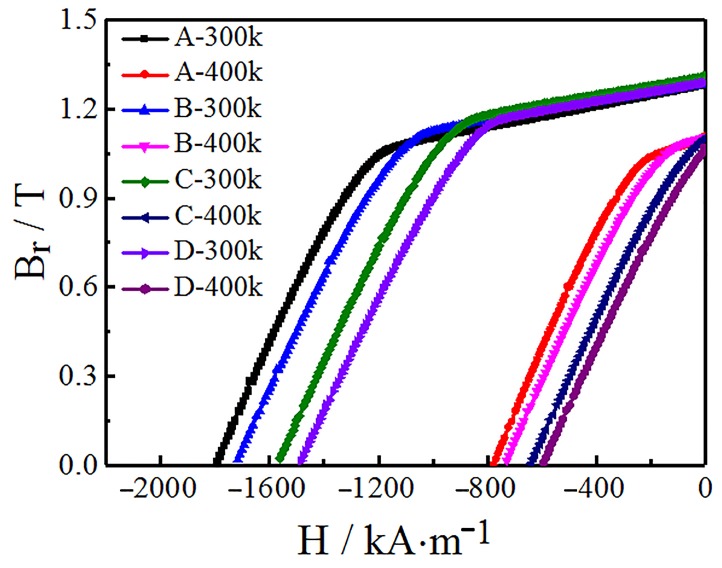
The variable temperature demagnetization curve of different regions of diffusion magnets at 300 K and 400 K.

According to Figure 3, the demagnetization curve of different regions of diffusion magnets (locations A-D) shows that the variation rule of magnetic properties at 400 K are consistent with that at 300 K. In other words, the increase of diffusion depth does not significantly change *B*_r_ and (*BH*)_max_. It can also combine with the reversible temperature coefficient formula of *B*_r_ and *H*_cj_ [16], namely Equations (1) and (2):

(1) $$\alpha_{B_{r}}=\frac{B_{r}(T)-B_{r}(T_{0})}{B_{r}(T_{0})(T-T_{0})}$$

(2) $$\beta_{H_{\mathrm{cj}}}=\frac{H_{\mathrm{cj}}(T)-H_{\mathrm{cj}}(T_{0})}{H_{\mathrm{cj}}(T_{0})(T-T_{0})}$$

where *α**_B_*****_r_** is the *B*r temperature coefficients, *β_H_***_cj_** is the *H***_cj_** temperature coefficients, *T*_0_ stands for the room temperature, *T* is the testing temperature, and *B*_r_*(T)*, *B*_r_*(T***_0_***)* and *H*_cj_*(T)*, *H*_cj_*(T*_0_*)* represent the *B***_r_** and *H***_cj,_** at different temperatures, respectively.

In the temperature range of 300 K to 400 K, the *B*r temperature coefficients (*α**_B_*****_r_**) and the *H*_cj_ temperature coefficients (*β_H_***_cj_**) under various diffusion depths of the magnet are shown in Table 2. This characterizes the deterioration of magnetic properties with temperature, and the smaller their value, the better the temperature stability. The above results show that the increase in diffusion depth caused a gradual increase in *α**_B_*****_r_** and *β_H_***_cj_**, which enhanced from 0.12% K^−1^ and 0.55% K^−1^ at the surface of the magnet (location A) to 0.16% K^−1^ and 0.59% K^−1^ at the center (location D), respectively. This indicates that the stability of the GBDP magnet varies with the diffusion depth. Better stability of the *B*r and *H*_cj_ was obtained closer to the magnet surface. The difference in stability mainly depended on the Tb element distribution, phase structure, and grain boundary morphology at each diffusion depth [17,18]. The diffusion of Tb led to the grain exchange decoupling and high-Ha shell formation, which resulted in the increase of α and decrease in the number of magnetic atoms per unit volume (Neff) [8]. Therefore the sample kept a higher thermal stability.

**Figure 4:**
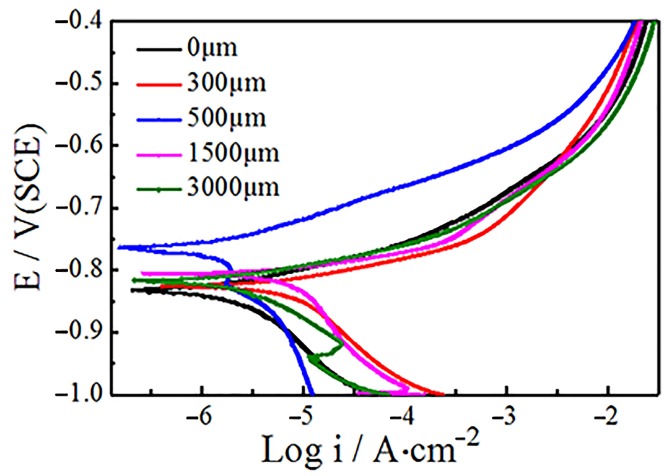
The Tafel curve at different diffusion depths of the magnets.

As shown in Figure 4, the Tafel curves of different depths of magnets were measured, and the influence of diffusion on the microstructure was analyzed through the change of the corrosion performance of the GBDP magnets at various positions. The test results show that the corrosion potential of the surface magnet was the lowest, only −0.831 V, with a large corrosion tendency. However, as the diffusion depth increased to 350 µm, the corrosion potential moved to the positive direction, and reached the maximum value of −0.764 V at the diffusion depth of 500 µm. The diffusion layer at the location of 500 µm had the least corrosion tendency and was not easy to be corroded. Subsequently, the increase of diffusion depth led to the decrease of potential and the corrosion performance, but the changes of potential and corrosion performance tended to be stable after 1500 µm. The above results also indicate that GBDP will cause various microstructure changes at different diffusion depths of magnets.

**Figure 5:**
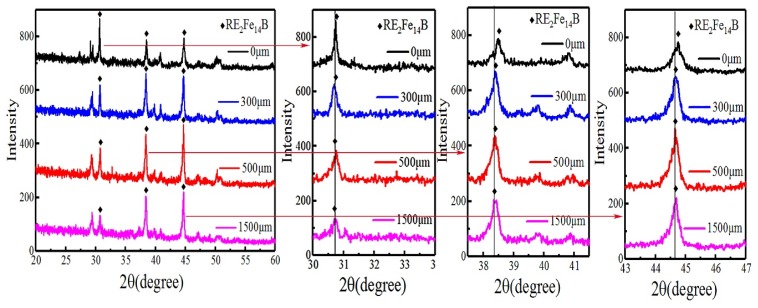
The XRD pattern of the magnet at different diffusion depth.

In order to further understand the relationship among the phase structure, microstructure, and performance of GBDP magnets, the structural information of different diffused magnets was first measured by XRD, as shown Figure 5. The calibration results of the diffraction peak showed that Tb diffusion would not produce a new phase in the magnet, but would lead to the structural changes of the main phase RE_2_Fe_14_B, as reported in a previous study [19]. With the depth of diffusion, the major diffraction peak at 31°, 41°, and 44.5° were shifted in the direction of a large angle, especially at the surface layer. Under the influence of a large Tb concentration gradient in the surface layer, Tb not only diffused along the grain boundary, but also diffused in the crystal to displace Nd in the 2:14:1 main phase. The radius of the Tb atom is 0.1782 nm smaller than the 0.2978 nm radius of Nd atom, which reduces the lattice constant of the host phase and shifts the diffraction peak to a larger angle [20]. With the increase of diffusion depth, the Tb concentration and the tendency of magnetic grain diffusion decreased. Therefore, the displacement of Tb elements into the 2:14:1 phase was reduced, and the diffraction peak movement was very small.

**Figure 6:**
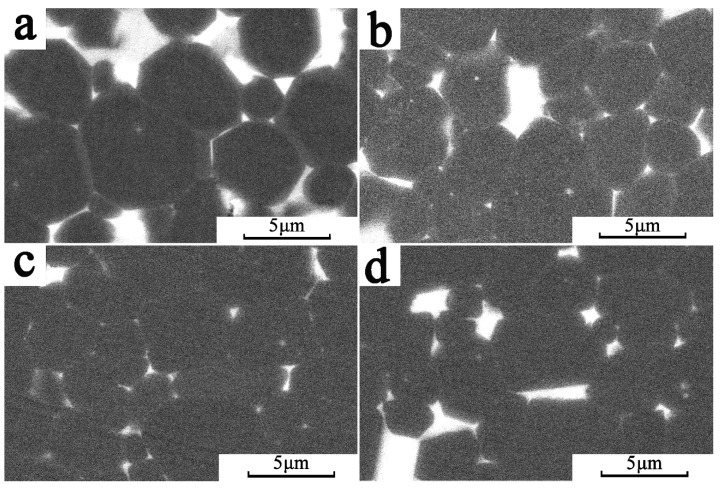
The SEM images of diffusion magnets: (**a**) surface BSE image; (**b**) 300 μm; (**c**) 500 μm; (**d**) 1500 μm.

Figure 6 shows the microstructure of the magnet at different diffusion depths characterized by SEM. During Tb_2_O_3_ diffusion, a large number of white RE-rich phases were agglomerated at the triangular grain boundary on the surface magnet. The GBDP of magnet reduced the ratio of the hard magnetic phase and made the *B*_r_ decline, as shown in Figure 6a. In addition, the light gray (Nd,Tb)_2_Fe_14_B core-shell structure, which led to the enhancement of local magnetocrystalline anisotropy field and finally caused the increase of *H*_cj_ and the deterioration of *B*_r_ and (*BH*)_max_, was observed outside the grain. With the increasing of diffusion depth, the agglomeration of RE-rich phase decreased at the diffusion depth of 300 μm, and was distributed uniformly and continuously around the main phase, as shown in Figure 6b. Moreover, the core-shell structure could still be observed in the main phase grains, but as its number decreased significantly [21], its effect on increasing the coercivity of magnets was weakened and the deterioration of *B*_r_ and (*BH*)_max_ was improved. A continuous grain boundary phase could efficiently avoid the exchange coupling of the main phase, and increase the *H*_cj_ of magnets. When the diffusion depth reached 500 μm, there was no rare-earth enrichment and core-shell structure, as shown in Figure 6c. The decrease in the core-shell structure and the RE-rich phase led to a change in the magnetic properties, the increase of *B*r, and the decrease of the *H*_cj_. In Figure 6d, the RE-rich phase in the magnet center (1500 μm) was minimal and discontinuous, which increased the magnetic exchange coupling of the main phase and further reduced *H*_cj_ compared with the surface part of the magnet. For the whole Tb diffusion magnets, the Tb atoms entered into the tetragonal Nd**_2_**Fe**_14_**B to enhance *H***_a_** and the improved microstructure was the main reason for the improvement of *H***_cj_**.

**Figure 7:**
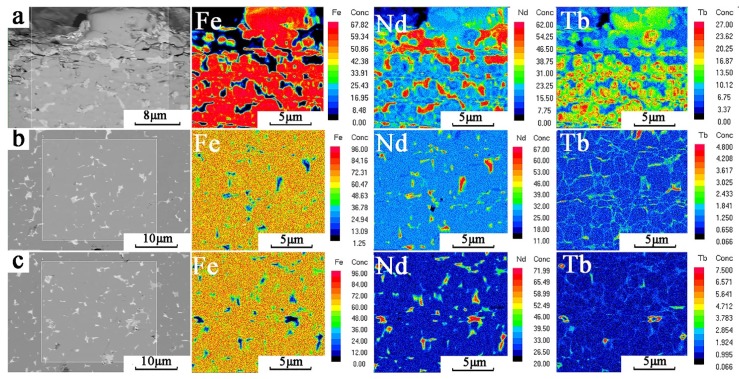
The EPMA images of the distribution of Fe, Nd and Tb at different depths, (**a**), (**b**), (**c**) are the SEM images at 0 μm, 500 μm, 1500 μm from the surface of the magnet.

Figure 7 shows the distribution of elements with different diffusion depths of magnets determined by EPMA. A large amount of high concentration Nd was enriched in the rare earth phase of the surface grain boundary, as shown in Figure 7a. The closer the distance to the surface layer, the higher the proportion of the surface ratio of the RE-rich phase. Tb and Fe were mainly distributed in the main phase, where Tb gathered in grain outer layer to form thick (Nd,Tb)_2_Fe_14_B core-shell structures. Under the influence of the concentration gradient, Tb diffused and replaced Nd in the main phase, which is an important reason for the enrichment of Nd in the grain boundary and Tb in the outer layer. The above results are also consistent with the SEM images in Figure 6a.

Figure 7b shows the element distribution of the GBDP magnet at the diffusion depth of 500 μm. The results prove that Fe and Nd are distributed in the main phase and RE-rich phase, respectively. However, the distribution of RE-rich phases was different to that of the surface layer, which distributed in a discontinuous form around the RE_2_Fe_14_B grains and decreased in proportion. Distribution of Tb was obviously different from that of surface layer. Some Tb locations coincide with Nd locations, indicating that Tb entered the RE-rich phase. The remaining Tb was in the opposite position to Nd, and appeared in the outer edge of the main phase grain to form a very thin core-shell structure.

As shown in Figure 7c, the Tb-rich core-shell structure at the outer edge layer of the main phase grain disappeared as the diffusion depth reached 1500 μm. Tb mainly agglomerated with Nd in the RE-rich phase at the triangular grain boundary. The proportion of the rare earth phase increased slightly compared with that at 500 μm, but the continuity became worse.

The element diffusion process is an important factor affecting the grain boundary diffusion effect according to the Second Fick Law [22], see Equation (3):

(3) $$C(x,t)=\frac{M}{2\sqrt{\Pi Dt}}\exp(-\frac{x^{2}}{4Dt})$$

where *C* stands for the element concentration, *M* is the Mass per unit area, *D* represents the diffusion coefficient, *x* is the diffusion depth, and *t* is the diffusion time.

The diffusion depth and rate of elements were changed by the diffusion coefficient *D*, which is closely related to the concentration of diffusion elements, medium state, structure, and micro-defects. The higher the concentration of elements, the greater the diffusion coefficient and diffusion tendency. At the same temperature, the diffusion activation energy of the melted RE-rich phase was much lower than that of the solid phase [23]. Therefore, the RE-rich phase also showed a larger diffusion coefficient than the main phase. Compared with the close-packed crystal structure of the Nd_2_Fe_14_B, the HCP of the RE-rich phase had a simple structure and relatively loose atomic arrangement, which was more conducive to diffusion and exhibited a larger diffusion coefficient [22]. In addition, the low coherence, poor interfacial stability, and numerous micro-defects of different adjacent phases also led to the high diffusion coefficient of the grain boundary phase. In short, the energy required for intergranular diffusion was lower than that for intragranular diffusion.

**Figure 8:**
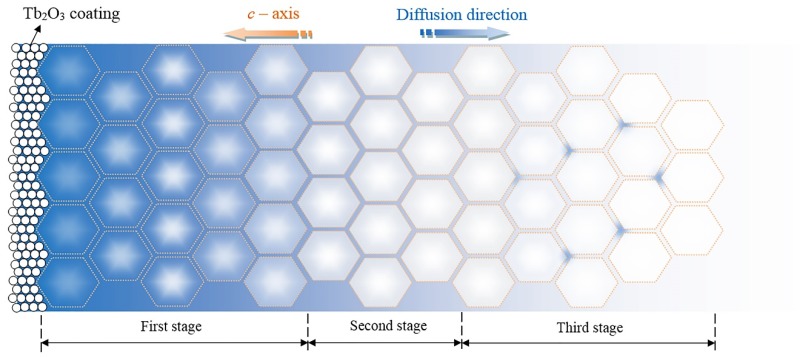
The diffusion path of the element at the diffusion depth of the magnet.

According to the above diffusion theory, combined with the EPMA and SEM test results, the Tb diffusion path and the causes of the microstructures in different depths can be obtained, and the plane diffusion schematic diagram under concentration gradient can be constructed, as shown in Figure 8. In Figure 8, the hexagonal regions represent the main phase, and the pores between them represent the grain boundaries, the blue areas in the figure represent the approximate distribution of Tb in the GBDP magnets. The spread process of Tb can be divided into three stages. 

The first stage is located on the surface of the magnet, with a significant difference in Tb concentration and a large diffusion driving force. Tb diffusion is preferentially carried out along the grain boundaries, which greatly increases the proportion of the RE-rich phase of magnets. However, the limited grain boundary diffusion channel is still difficult to meet the demand of Tb diffusion along the depth direction. The high concentration gradient drives the Tb diffusion driving force to exceed the intragranular diffusion activation energy, resulting in an intragranular diffusion. Nd is partially replaced by Tb in the Nd_2_Fe_14_B main phase to form an image observed in EMPA (Figure 7a). The formation of a thick core-shell structure layer in the main phase epitaxy layer increases the local magnetocrystalline anisotropy and the coercivity. However, the increase of RE ratio and Tb content in the main phase also results in the decrease of surface *B*_r_ and (*BH*)_max_, as shown in Figure 2.

The second stage is located at 300–1000 μm, in the subsurface layer of the magnet, where the Tb concentration decreases dramatically. Therefore Tb diffusion is dominated by the intergranular diffusion, and the intragranular diffusion behavior is weakened. Only a little Tb diffuses into the main phase to form a thin core-shell layer, which enhances the local magnetocrystalline anisotropy, as shown in Figure 7b. More Tb enriches at the grain boundaries to form homogeneous and continuous RE-rich phases, which prevents the magnetic exchange coupling between the main phases and plays an important role in the coercivity. 

The third stage is located in the deeper diffusion position of the magnet. With the further decrease of element concentration, Tb neither diffuses into the main phase nor forms a continuous RE-rich phase, but instead, diffuses at the triangular grain boundary, as shown in Figure 7c. This phenomenon improves the wettability of the grain boundary phase and promotes the continuous distribution of RE-rich phases [24], but the effect of *H*_cj_ is less than that of the first and second stages.

The diffusion of Tb also explains the corrosion behavior of magnets at different depths. Diffusion increases the proportion of the RE-rich phase in the surface layer of the magnet, which leads to the increase of the surface defects (as show in Figure 7a) and the decrease of the corrosion resistance. The increase of diffusion depth decreases the grain boundary ratio, while the enrichment of Tb in the rare earth phase improves the wettability of the grain boundary and reduces the number of defects that reduces the potential difference between the main phase and the RE-rich phase, which greatly improves the corrosion resistance [25,26]. With the further increase of diffusion depth, the Tb content at grain boundary decreases, and its corrosion resistance decreases.

## 4. Conclusions

The GBDP of Tb_2_O_3_ has a significant enhancement effect on the *H*_cj_ of magnets. However, with the increase of diffusion depth, the coercivity increment decreases gradually, and the temperature stability of the remanence and coercivity becomes worse. These are due to the three stages of element diffusion. The first stage is located on the surface of the magnet, forming a thick (Nd,Tb)_2_Fe_14_B core-shell structure and a relatively high proportion of the RE-rich phase, which could significantly enhance the coercivity. The second stage is located in the sub-surface layer, forming a continuous RE-rich phase and thin Tb-rich core-shell structure layer, which reduced the increment of coercivity. The third stage is located deeper in the magnet, and only a small amount of Tb is concentrated at the triangular grain boundary, which resulted in a slight decline in coercivity.

Tb_2_O_3_ diffusion also causes corrosion resistance differences at various locations. The high RE-rich ratio at the surface of the magnet leads to many surface defects and poor corrosion resistance. Tb on the subsurface layer of magnet could reduce the phase potential difference between the main phase and the RE-rich phase, which leads to the decrease of corrosion tendency. However, with the increase of diffusion depth, the Tb concentration at the grain boundary decreases and the corrosion resistance becomes worse.

## Figures and Tables

**Table 1:** The changes of magnetic properties at 300 K after Tb_2_O_3_ diffusion.

Materials	B_r_/T	H_cj_/KA·m^−1^	(BH)_max_/kJ·m^−3^
Original magnets	1.407	1180.17	351.48
GBDP magnets	1.395	1685.13	347.98

**Table 2:** The *α**_B_*****_r_** and the *β_H_***_cj_** under various diffusion depth of magnet in the range of 300–400 K.

Temperature Coefficients	A	B	C	D
*α_B_*_r_/% K^−1^	0.12147	0.13030	0.14169	0.16183
*β_H_*_cj_/% K^−1^	0.55635	0.57208	0.59032	0.59818
